# The role of chemotherapy in patients with T1bN0M0 triple-negative breast cancer: a real-world competing risk analysis

**DOI:** 10.7150/jca.52540

**Published:** 2021-01-01

**Authors:** Tian Lan, Yunyan Lu, Hua Luo, Ouou Yang, Junling He, Haibin Xu, Zujian Hu

**Affiliations:** 1 Department of Breast Surgery, Guangxing Hospital Affiliated to Zhejiang Traditional Chinese Medicine University, Hangzhou Hospital of Traditional Chinese Medicine, Hangzhou, Zhejiang, People's Republic of China; 2 The Second Clinical Medical College, Zhejiang Chinese Medical University, Hangzhou, Zhejiang, People's Republic of China; 3 Department of Cardiology, The First People's Hospital of Xiaoshan District, Hangzhou, Zhejiang, People's Republic of China; Tian Lan and Yunyan Lu contributed equally to this work.

**Keywords:** triple-negative breast cancer, chemotherapy, T1bN0M0 breast cancer, survival analysis

## Abstract

The objective of the present study was to implement Kaplan-Meier analysis, competing risk analysis, and propensity score matching to evaluate whether the patients with T1bN0M0 triple-negative breast (TNBC) could benefit from adjuvant chemotherapy. A total of 1849 patients were identified in the Surveillance, Epidemiology, and End Results (SEER) database from 2010 to 2015. All eligible patients were divided into two cohorts, the chemotherapy (1155 patients) and the no-chemotherapy (694 patients) cohorts. Similar 5-year breast cancer-specific survival (BCSS) was observed in the chemotherapy and no-chemotherapy cohorts (96.1% vs. 96.0%, p=0.820). The results of the competing risk analysis showed a comparable 5-year breast cancer-specific death (BCSD) in both groups (chemotherapy 3.6% vs. no-chemotherapy 3.4%, p=0.778). Also, a higher 5-year other causes death (OCD) was observed in the no-chemotherapy cohort (0.7% vs. 5.4%, p<0.001). Multivariable competing risks regression models showed no association between chemotherapy and BCSS (HR, 1.21; 95%CI, 0.64-2.31; p=0.560). After 1:1 PSM, no significant difference was also observed for BCSD and OCD between two cohorts. The value of adjuvant chemotherapy in patients with T1bN0M0 TNBC is less than the present guidelines recommend, suggesting that de-escalated treatment could be a potentially beneficial strategy in appropriately selected patients.

## Introduction

Breast cancer is one of the most common malignancies in women worldwide, with more than 250,000 new cases reported during 2020 1. It is a highly heterogeneous disease that can be divided into four subtypes (luminal A, luminal B, Her2-enriched, and triple-negative) based on the expression of estrogen receptor (ER), progesterone receptor (PR), human epidermal growth factor 2 (HER2), and Ki67 2. Triple-negative breast cancer (TNBC, negative expression of ER, PR, and HER2), accounting for 15% of breast cancers all over the world, presents a worse prognosis and has different clinicopathologic features in comparison with other molecular subtypes 3, 4. In clinical practice, chemotherapy is the standard of treatment for TNBC due to a lack of effective target therapies. Clinicians usually choose tailored chemotherapy on the basis of TNM stage, tumor grade, Ki67, and performance status of each patient.

A rapid rise in the occurrence of breast cancer that is less than 1 cm has been attributed to the extensive implementation of ultrasound and mammogram 5. TNBC was reported as an independent risk predictor for prognosis in T1b breast cancer 6. Currently, the National Comprehensive Cancer Network (NCCN) guidelines recommend consideration of chemotherapy in patients with T1bN0M0 TNBC. However, these specific patients have a less than 10% risk of recurrence and may not benefit from adjuvant chemotherapy in some literature 7-9. Therefore, the benefit of adjuvant chemotherapy in patients with T1bN0M0 TNBC remains controversial.

In the present study, we conducted a retrospective, population-based study using the Surveillance, Epidemiology, and End Results (SEER) database in order to interpret the potential effect of chemotherapy in patients with T1bN0M0 TNBC.

## Methods

### Data source and patient selection

The SEER database (http://seer.cancer.gov/), which encompasses approximately 28% of the US population, was used in this retrospective study performing the SEER*Stat 8.3.5 software. As Her2 information has been recorded in the SEER database since 2010, patients diagnosed after 2010 were included in this study. The eligible patients were identified according to the following inclusion criteria: (1) female patients; (2) years of diagnosis from 2010 to 2015; (3) patients aged over 18; (4) breast cancer as the only malignancy; (5) infiltrating duct carcinoma (8500/3) confirmed by histological diagnosis; (6) triple-negative breast cancer (HR-/HER2-); (7) T1bN0M0; (8) patients who received surgery; (9) patients who survived more than one month. Patients with incomplete or missing clinicopathological data were excluded from the study. Ultimately, 1849 eligible patients were identified for further investigation.

### Study variables

Demographic data (age at diagnosis, marital status, ethnicity, and median household income), tumor grade, treatment regimens (radiotherapy and chemotherapy), and prognostic information were extracted from the SEER database. Patients were split into chemotherapy (patients who received chemotherapy) and no-chemotherapy (patients who did not receive chemotherapy) cohorts. Age was defined as a continuous variable. We classified the patients into two groups according to their ethnicity (white and nonwhite). Patients were divided into four groups according to the median household income: quartile 4 (>$74441), quartile 3 ($60891 - $74440), quartile 2 ($52621- $60890), and quartile 1 (<$52620).

### Statistical analyses

Differences in the baseline clinicopathological parameters between the chemotherapy and no-chemotherapy cohorts were determined using the t-test or the chi-squared test. We performed survival analyses of overall survival (OS) and breast cancer cause-specific survival (BCSS) by using the Kaplan-Meier analysis and determined the significant distinctions by employing the log-rank test. To accurately consider the effects of competing risks in the survival data, patients were tagged as three outcomes of interest: alive, breast cancer-specific death (BCSD), or other causes death (OCD). The effect of chemotherapy in T1bN0M0 TNBC patients was further evaluated by cumulative incidence function and the Fine and Grey's proportional subdistribution hazard model. These competing risk analyses were performed using the R package “cmprsk” 10.

The propensity score matching (PSM) method is a powerful tool for reducing the influences of confounding variables in retrospective studies 11 and is increasingly employed to simulate a randomized controlled trial scenario 12. Therefore, we utilized 1:1 PSM with a nearest-neighbor algorithm to re-examine the role of chemotherapy in the T1bN0M0 TNBC by using the “MatchIt” package 13. All statistical analyses and visualization were performed using R (version 3.5.2, https://www.r-project.org/). Two-sided p values less than 0.05 were defined as statistically significant.

## Results

### Patient characteristics

A total of 1849 patients with T1bN0M0 TNBC fulfilled the inclusion criteria in the SEER database from 2010 to 2015. Of these patients, 1155 patients had been treated with chemotherapy and 694 patients had not received chemotherapy. The detailed clinicopathological information of the patients was listed in **Table 1**. There were significant differences among age, marital status, and tumor grade between the chemotherapy and no-chemotherapy cohorts (all p<0.001). The patients with chemotherapy presented with a higher percentage of married (66.8% vs. 56.6%), lower age (mean age, 57.06 vs. 67.24), and higher grade (grade III, 75.6% vs. 62.4%) in comparison with those without chemotherapy. Meanwhile, the composition ratio of median household income showed a tendency of difference between two cohorts (p=0.058). No differences were observed in radiotherapy usage between two cohorts.

### Survival analysis

**Figure 1:**
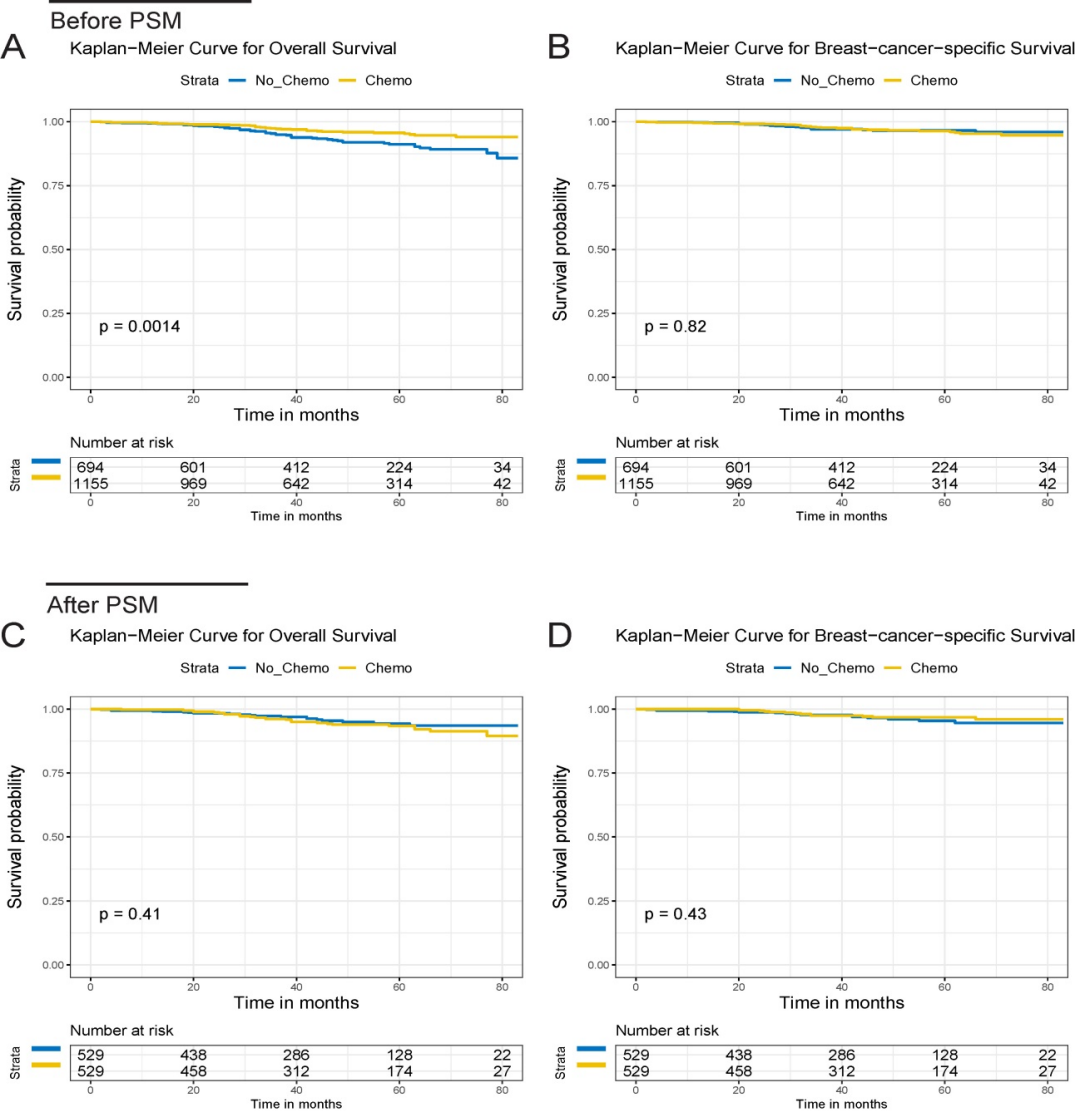
OS and BCSS of the T1bN0M0 triple-negative breast cancer patients for the chemotherapy and no chemotherapy cohorts. OS (A) and BCSS (B) curves before PSM. OS (C) and BCSS (D) curves after PSM.

**Figure 2:**
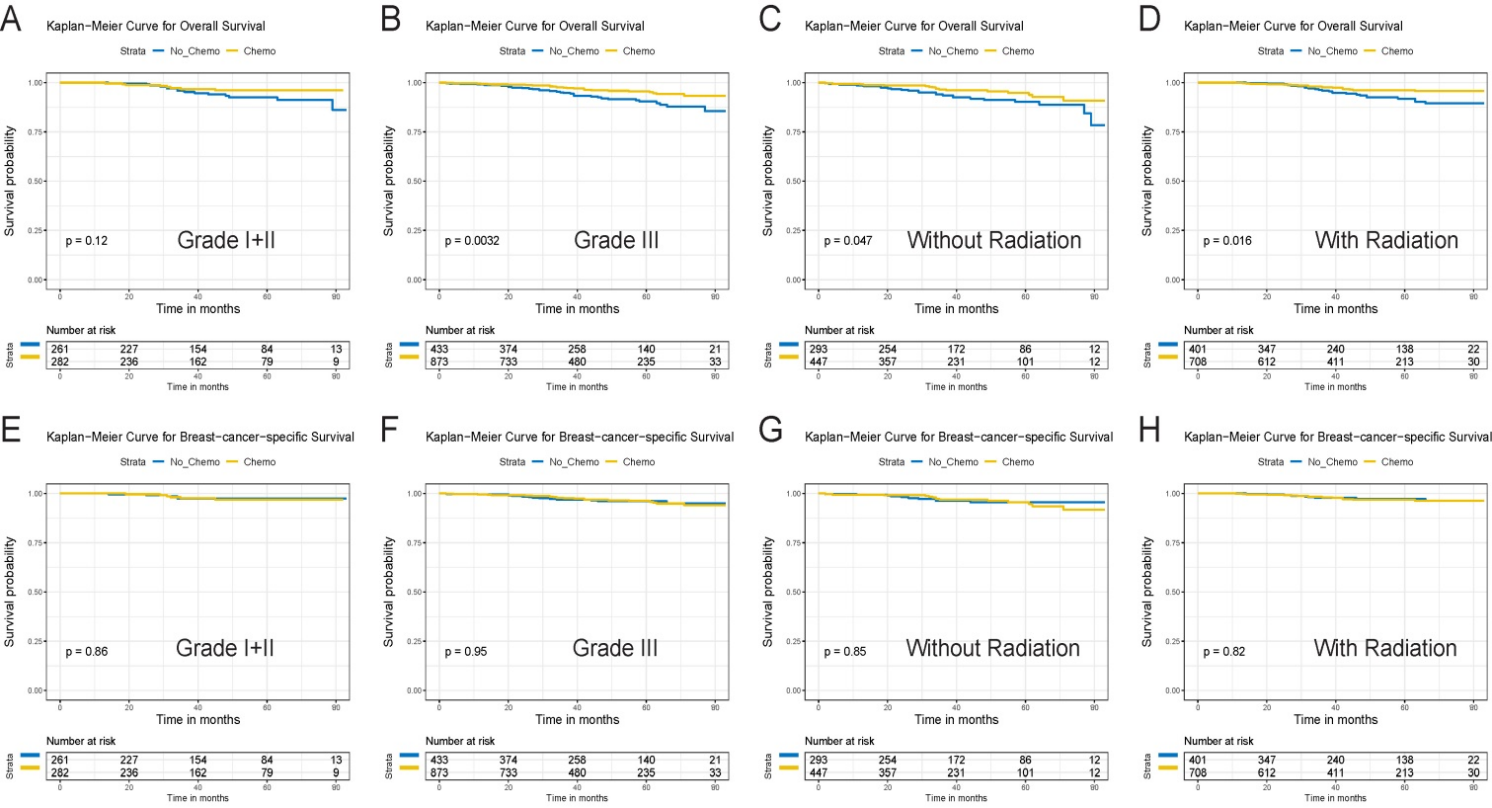
Survival curves for the T1bN0M0 triple-negative breast cancer patients for the chemotherapy and no chemotherapy cohorts in different subgroups before PSM. OS (A) and BCSS (E) in grade I+II cohort. OS (B)and BCSS (F) in grade III cohort. OS (C) and BCSS (G) in no radiation cohort. OS (D) and BCSS (H) in radiation cohort

After a median follow-up of 44 months (ranging from 2 to 83 months, 43 and 46 months in the chemo and no-chemo cohorts), 86 deaths were observed in this study (32 BCSD and 6 OCD in the chemotherapy cohort, 19 BCSD and 29 OCD in the no-chemotherapy cohort). 5-year OS was 95.4% in patients with chemotherapy versus 90.2% in those without chemotherapy (p=0.001,** Figure 1A**). There were similar 5-year BCSS in the chemotherapy and no-chemotherapy cohorts (96.1% vs. 96.0%, p=0.820,** Figure 1B**). Subgroup analyses based on tumor grade and radiotherapy revealed that 5-year OS in the chemotherapy cohort was still significantly higher than that in the no-chemotherapy cohort except in the grade I+II subgroup (**Figure 2A**-**D**). However, no significant differences were observed in BCSS between the chemotherapy and no-chemotherapy cohorts regardless of grade (**Figure 2E** and **2F**) and radiotherapy (**Figure 2G** and** 2H**). Besides, the results of the univariate Cox analyses, which was similar to that of the Kaplan-Meier analyses, revealed that chemotherapy was linked with improved OS (HR, 0.51; 95%CI, 0.33-0.78; p=0.002) (**Table S1**), but not BCSS (HR, 1.07; 95%CI, 0.61-1.88; p=0.821) (**Table S2**).

**Figure 3:**
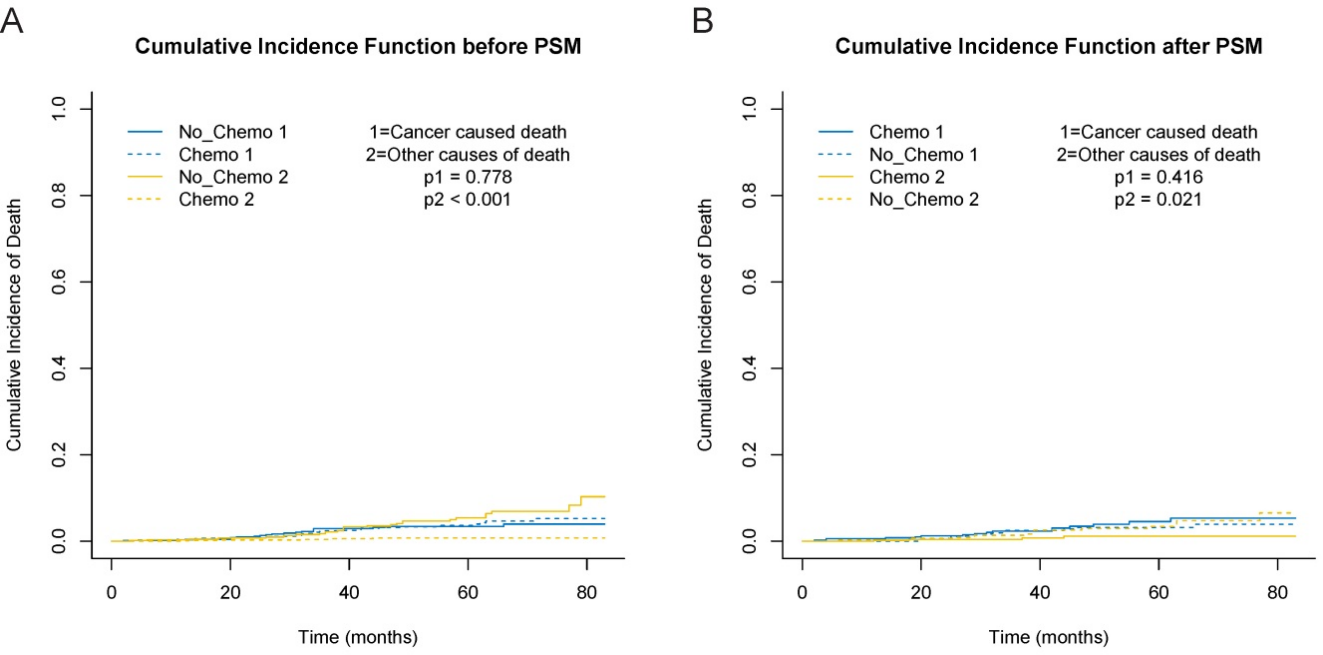
Cumulative incidence curves depicting breast cancer specific deaths and deaths from other causes before and after PSM. A, cumulative incidence curves before PSM. B, cumulative incidence curves after PSM.

**Figure 4:**
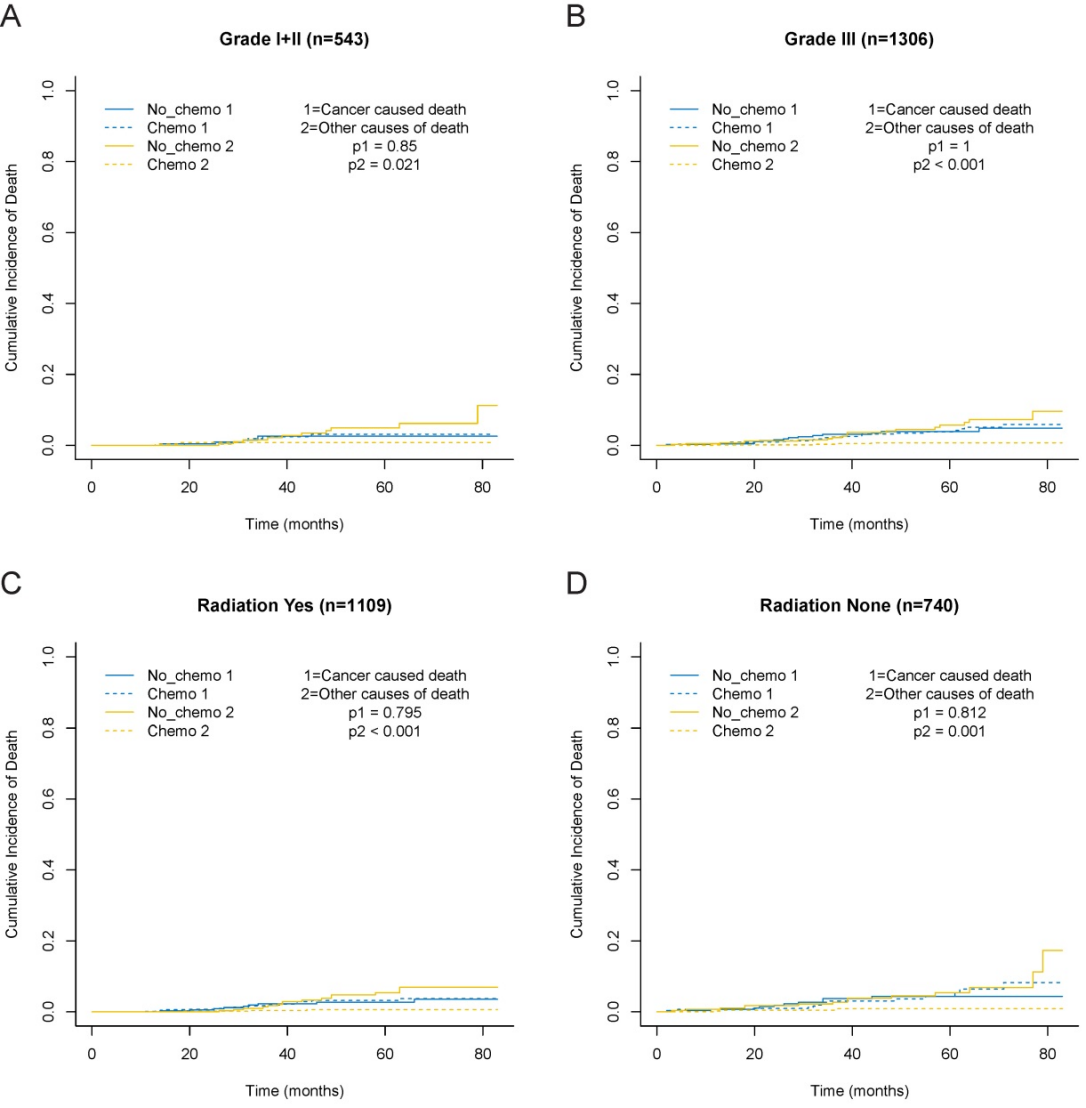
Cumulative incidence curves for the T1bN0M0 triple-negative breast cancer patients according to chemotherapy in different subgroups based on before PSM. A, grade I+II cohort. B, grade III cohort. C, no radiation cohort. D, radiation cohort.

In consideration of competing risks (death from other causes), we further performed cumulative incidence plots in the overall population, indicating comparable 5-year cumulative incidence of BCSD in the chemotherapy and no-chemotherapy groups (3.6% vs. 3.4%, p=0.778) and a higher 5-year cumulative incidence of OCD in the no-chemotherapy cohort (0.7% vs. 5.4%, p<0.001) (**Figure 3A, Table S3**). In the subgroup analyses focused on grade (**Figure 4A** and **4B**) and radiotherapy (**Figure 4C** and **4D**), the results were consistent with the findings in the overall population. Furthermore, there was no association between chemotherapy and BCSD in the multivariable competing risks regression models based on the overall cohort (HR, 1.33; 95%CI, 0.64-2.78; p=0.450) (**Table 2**).

After 1:1 PSM, the standardized difference (SD) of all baseline features were less than 0.1, which indicated a good agreement between the chemotherapy and no-chemotherapy groups (**Figure S1**). In addition, the clinicopathological characteristics after PSM were summarized in **Table 1**. After this approach, we observed that chemotherapy did not decrease BCSD and OCD in patients with T1bN0M0 TNBC (**Figure 3B**). The 5-year cumulative incidences of BCSD were 4.5% and 3.2%, respectively, in the chemotherapy and no-chemotherapy cohorts (p=0.416). Meanwhile, a significant difference was observed for the 5-year cumulative incidence of OCD between the respective cohorts (1.1% vs. 3.4%, p=0.021) (**Table S3**).

## Discussion

In this study, we identified 1849 eligible patients from the SEER database and demonstrated that patients receiving chemotherapy could derive significant gain of OS, but not BCSS. Cancer-specific survival was reported as a more accurate endpoint compared to OS when investigating the real effect of chemotherapy because OS can be diluted by OCD 14. Moreover, the occurrence of OCD may hinder the observation of BCSD, particularly in breast cancer patients with relatively long survival times 15, 16. Given the existence of the competing risks that should not be censored and ignored, the competing risk analyses were implemented in the present study. By this procedure, we found that although OCD in the chemotherapy cohort was lower than that in the no-chemotherapy cohort, there was no difference in BCSD between the two cohorts. Patients who had received chemotherapy had some unfavorable pathophysiological features, such as higher tumor grade and younger age, which were consistent with previous studies 17, 18. Hence, the discrepancy of OCD between two groups may partially be explained by a higher proportion of patients with older age and unmarried status in the no-chemotherapy cohort, and some potential comorbidities unavailable in the SEER database 12, 19, 20. Subsequently, we performed further PSM analysis due to unmatched baseline features between the two cohorts. The findings after PSM were similar to the previous results, confirming that chemotherapy was not associated with BCSD in these specific patients.

According to the NCCN guidelines, chemotherapy should be considered for patients with T1bN0M0 TNBC. 77.6% of the St. Gallen panelists preferred anthracycline-free regimens (alkylating agent and taxane) for these patients 21. There were several retrospective studies concentrating on the small TNBC because of the exclusion of these patients in most prospective randomized clinical trials. Almost all previous researches have focused on T1 (T1a/b, T1b/c, T1a/b/c) TNBC 7, 17, 22-24. One recent study (including 45 TNBC) by Jean et al. revealed that chemotherapy was not associated with OS and disease-free survival (DFS) in patients with ≤1cm node-negative TNBC 23. Another retrospective study, which included 82 TNBC cases (some of them were positive node), suggested that there was a tendency for OS benefit from chemotherapy for these specific patients 24. Chemotherapy failed to provide any benefits for locoregional recurrence-free survival, metastasis-free survival, and DFS in two retrospective studies, including 174 T1a/b TNBC and 278 T1a/b TNBC patients, respectively 17, 18. Furthermore, Ren et al. observed improved recurrence-free survival from chemotherapy in T1c TNBC but not in T1b 7. Taken together, the limited sample size of T1bN0M0 TNBC and the different endpoints used in previous studies have made it difficult to identify the exact effects of chemotherapy in patients with T1bN0M0 TNBC. To our best knowledge, this is the first study to demonstrate the influence of adjuvant chemotherapy in T1bN0M0 TNBC patients and to assess the feasibility of de-escalated chemotherapy in the specific patients by using PSM and competing risk analysis.

In the conventional view, T1b TNBC breast cancer patients experience excellent outcomes with a low 5-year distant relapse of 4%-10% and high OS of 95% 9, 23. The T1bN0M0 TNBC also fared well overall in our study. The 5-year BCSD were the same both before and after PSM (3.6% for the chemotherapy group and 3.4% for the no-chemotherapy group). Besides, chemotherapy was not associated with improved BCSS (chemotherapy vs. no-chemotherapy, 96.1% vs. 96.0%).

In clinical practice, high-risk clinicopathological parameters (higher grade and higher Ki67) usually lead to chemotherapy administration. No BCSS benefit from chemotherapy was observed in the subgroup analysis based on tumor grade. Despite the limited value of chemotherapy in the current study, a certain subgroup of these patients who may benefit from chemotherapy should be identified accurately. Zhao and colleagues revealed that TNBC is a heterogeneous disease encompassing four molecular subtypes, such as luminal androgen receptor (LAR), immunomodulatory (IM), basal-like and immune-suppressed (BLIS), and mesenchymal-like (MES) 25. A precision medicine strategy based on this molecular classification may be an effective and viable tool to predict the role of chemotherapy in T1bN0M0 TNBC. Additionally, we maybe could use genomic and transcriptomic features to screen out lower-risk patients with T1bN0M0 TNBC who do not need chemotherapy in the future, like the 21-gene assay and the 70-gene assay which have been applied to tailor chemotherapy and to predict the prognosis in women with early hormone receptor-positive breast cancer 26, 27.

Our study adds further evidence for the use of chemotherapy in TNBC, yet some limitations of the current study should be noted. First, some important clinical and genomic data relating to T1bN0M0 TNBC, including Ki67, lymphovascular invasion, BRCA1/2 status, detailed lymph node status (isolated tumor cells or micrometastases), and TNBC molecular classification, are unavailable in the SEER database. The lack of detailed chemotherapy information, such as dose, regimen, and adverse effects, also makes it difficult to interpret the comprehensive influences of different chemotherapy regimens. Second, a longer follow-up duration is needed to validate our results. The information associated with recurrence was not collected in the SEER database, which made it tough to compare with other previous studies. OS, BCSS, BCSD, and OCD recorded in the SEER database were defined as the primary endpoints of our study. Third, selection bias was inevitable as our study was performed retrospectively. Despite the difficult recruitment of these specific patients, further prospective randomized control trials should be warranted.

In conclusion, this was a retrospective real-world study to describe the numbers of BCSD and OCD in T1bN0M0 TNBC. By applying competing risk analysis and PSM, we demonstrated that these specific patients had no benefit of BCSS from chemotherapy. The value of chemotherapy is less than the present guidelines recommend. De-escalation of chemotherapy should be considered for certain patients with T1bN0M0 TNBC.

## Supplementary Material

Supplementary figure and tables.

## Figures and Tables

**Table 1:** The descriptive characteristics of T1bN0M0 TNBC before and after PSM

	Before PSM			After PSM		
Characteristics	No_Chemo	Chemo	P value	No_Chemo	Chemo	P value
	694	1155		529	529	
Age (mean (SD))	67.24(11.71)	57.06(10.29)	<0.001	63.38(10.42)	62.82(9.18)	0.356
Marital_status (%)						
Married	393(56.6)	772(66.8)	<0.001	335(63.3)	327(61.8)	0.657
Unmarried	301(43.4)	383(33.2)		194(36.7)	202(38.2)	
Race (%)						
White	537(77.4)	871(75.4)	0.366	407(76.9)	389(73.5)	0.226
Nonwhite	157(22.6)	284(24.6)		122(23.1)	140(26.5)	
Median_household_income (%)						
Quartile 1	211(30.4)	375(32.5)	0.058	162(30.6)	156(29.5)	0.929
Quartile 2	181(26.1)	241(20.9)		140(26.5)	138(26.1)	
Quartile 3	166(23.9)	279(24.2)		121(22.9)	130(24.6)	
Quartile 4	136(19.6)	260(22.5)		106(20.0)	105(19.8)	
Grade (%)						
I+II	261(37.6)	282(24.4)	<0.001	172(32.5)	173(32.7)	1.000
III	433(62.4)	873(75.6)		357(67.5)	356(67.3)	
Radiation (%)						
None	293(42.2)	447(38.7)	0.148	203(38.4)	226(42.7)	0.168
Yes	401(57.8)	708(61.3)		326(61.6)	303(57.3)	

SD, standard deviation.

**Table 2:** The results of multivariate subdistribution hazard model for T1bN0M0 TNBC

	Before PSM			After PSM		
Characteristics	HR	95%CI	P value	HR	95%CI	P value
Age	1.01	0.98-1.04	0.48	1.00	0.97-1.03	0.81
Marital_status						
Married	Reference			Reference		
Unmarried	1.21	0.71-2.08	0.49	1.38	0.69-2.78	0.36
Race						
White	Reference			Reference		
Nonwhite	1.22	0.67-2.20	0.52	1.32	0.60-2.91	0.49
Median_household_income						
Quartile 1	Reference			Reference		
Quartile 2	0.71	0.39-1.28	0.25	0.66	0.31-1.39	0.28
Quartile 3	0.93	0.51-1.68	0.81	1.19	0.54-2.64	0.67
Quartile 4	0.81	0.46-1.44	0.48	1.20	0.55-2.63	0.64
Grade						
I+II	Reference			Reference		
III	1.53	0.78-3.00	0.22	1.08	0.48-2.40	0.86
Radiation						
None	Reference			Reference		
Yes	0.63	0.36-1.09	0.10	0.57	0.27-1.19	0.14
Chemotherapy						
None	Reference			Reference		
Yes	1.21	0.64-2.31	0.56	1.33	0.64-2.78	0.45

HR, hazard rate; CI, confidence interval.

## Abbreviations

SEER: Surveillance, Epidemiology, and End Results
BCSS: breast cancer-specific survival
BCSD: breast cancer-specific death
BLIS: basal-like and immune-suppressed
HR: hazard ratio
IM: immunomodulatory
LAR: luminal androgen receptor
MES: mesenchymal-like
OCD: other causes death
PSM: propensity score matching
SD: standardized difference
TNBC: triple-negative breast
