# Supplementary material for: The role of chemotherapy in patients with T1bN0M0 triple-negative breast cancer: a real-world competing risk analysis
Source: J Cancer. 2021 Jan 1;12(1):10–7. doi: 10.7150/jca.52540 (PMC7738821; doi:10.7150/jca.52540)
Supplement: Supplementary file 1 — Supplementary figure and tables. [file jcav12p0010s1.pdf]

Table S1. OS in univariate analysis

Characteristics	HR	95%CI	P value
Age	1.05	1.03-1.07	0.00
Marital_status			
Married	Reference		
Unmarried	1.55	1.02-2.37	0.04
Race			
White	Reference		
Nonwhite	1.30	0.82-2.07	0.27
Median_household_income			
Quartile 1	Reference		
Quartile 2	0.79	0.52-1.19	0.27
Quartile 3	1.34	0.86-2.09	0.19
Quartile 4	0.91	0.57-1.45	0.68
Grade			
I+II	Reference		
III	1.16	0.72-1.88	0.53
Radiation			
None	Reference		
Yes	0.64	0.42-0.98	0.04
Chemotherapy			
None	Reference		
Yes	0.51	0.33-0.78	0.00

HR, hazard rate; CI, confidence interval.

Table S2. BCSS in univariate analysis

Characteristics	HR	95%CI	P value
Age	1.01	0.98-1.03	0.61
Marital_status			
Married	Reference		
Unmarried	1.30	0.75-2.26	0.36
Race			
White	Reference		
Nonwhite	1.32	0.72-2.41	0.37
Median_household_income			
Quartile 1	Reference		
Quartile 2	0.70	0.39-1.25	0.22
Quartile 3	0.95	0.53-1.68	0.85
Quartile 4	0.83	0.47-1.46	0.51
Grade			
I+II	Reference		
III	1.54	0.79-3.00	0.20
Radiation			
None	Reference		
Yes	0.64	0.37-1.12	0.12
Chemotherapy			
None	Reference		
Yes	1.07	0.61-1.88	0.82

HR, hazard rate; CI, confidence interval.

Table S3. The effect of chemotherapy for T1bN0M0 TNBC by cumulative incidence function analysis before and after PSM

	5-year CID of cancer	P value	5-year CID of other	P value
Before PSM				
Chemo	0.036	0.778	0.007	<0.001
No_Chemo	0.034		0.054	
After PSM				
Chemo	0.045	0.416	0.011	0.021
No_Chemo	0.032		0.034	

CID, cumulative incidences of death.

Figure S1

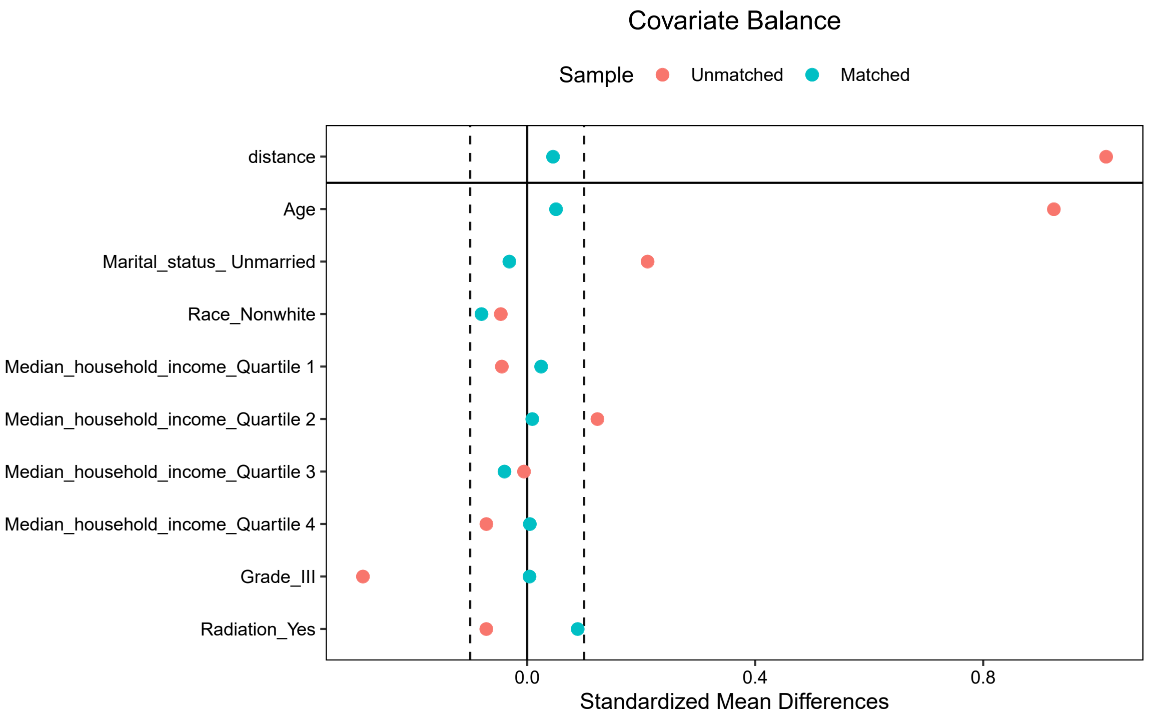
Covariate Balance
Sample
Unmatched
Matched
distance
Age
Marital_status_ Unmarried
Race_Nonwhite
Median_household_income_Quartile 1
Median_household_income_Quartile 2
Median_household_income_Quartile 3
Median_household_income_Quartile 4
Grade_III
Radiation_Yes
0.0
0.4
0.8
Standardized Mean Differences
